# Response surface optimization of cupcake physicochemical and sensory attributes during storage period: Effect of apricot kernel flour addition

**DOI:** 10.1002/fsn3.2688

**Published:** 2021-12-20

**Authors:** Fahimeh Ahmadi, Narjes Aghajani, Ashraf Gohari Ardabili

**Affiliations:** ^1^ Department of Food Science and Technology Bahar Faculty of Food Science and Technology Bu‐Ali Sina University Hamadan Iran

**Keywords:** apricot kernel flour, color, cupcake, sensory attributes

## Abstract

Today, the demand for functional products has increased. Apricot kernel is an important source of protein, oil, and fiber and has high antioxidant and antimicrobial properties. In this study, the effect of adding different levels (15% and 30%) of apricot kernel flour (AKF) to the cupcake formulation on the physicochemical, textural, and sensory attributes of the produced cupcake during 14 days of storage was investigated and optimized by the response surface methodology (RSM) to find the optimum cupcake production with respect to maintaining the quality attributes of produced cake during storage period compared with control sample. The results showed that increasing AKF significantly increased the consistency and apparent viscosity of the dough, as well as the volume, height, and percentage of cake baking loss, but the moisture content and hardness of the cake did not show a significant difference compared with the control sample. Also, the crust and crumb color of the samples containing AKF were significantly lighter than the control sample. The results of optimization process showed that addition up to 30% AKF improved the sensory properties such as the crust and crumb color, texture, porosity, aroma, taste, and overall acceptance compared with the control sample. Samples containing 30% AKF were selected as the best formulation by panelists.

## Graphical abstract

The addition of AKF to cupcake formulation increased the viscosity, specific gravity, and consistency of the dough.

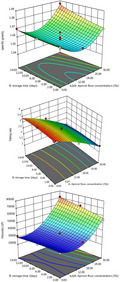

## 1 INTRODUCTION

Nowadays, due to consumers' interest in nutritional characteristics of foodstuffs, requests for functional and health‐giving foods have increased and the food industry has focused on redesigning of traditional foods to optimize nutritional value along with preserving or improving the taste of the product (Daraei Garmakhany et al., 2021). Bakery products are one of the most widely used food products in the world. Among these products, cake is welcomed and liked by consumers due to its good sensory properties, but due to its high sugar and fat, continuous and long‐term consumption of this food causes obesity and subsequent health problems; therefore, experts recommend low consumption of cake in diets. Therefore, by enrichment and improving the nutritional value of cake, a healthier product can be marketed. The definition of cake varies in different parts of the world, but the term cake refers to products made by formulations based on wheat flour, sugar, eggs, and liquids (such as milk and water), and fat or oil may also be added to their formulations. The amount of liquids added to the cake formulation is so high that a dough with low apparent viscosity is obtained compared with the bread dough. The sensory properties and shelf life of cakes vary according to their formulations.

The selection of suitable additives in different bakery products depends on various factors such as how additives affect the functional and rheological properties of the product and its cost effectiveness. The modification and manipulation of raw materials to improve the quality and increase the shelf life of food products are a powerful tool in the hands of manufacturers (Rodríguez‐García et al., 2014). Therefore, many works have been conducted in the field of replacement and modification of each component of cake formulation with the aim of enriching and improving the nutritional value or production of functional and pharmaceutical products for patients with certain diseases such as celiac disease. Examples include egg replacement with soy protein isolate in cake formulations with the aim of reducing cholesterol and allergens (Cauvain, 2003), producing low‐fat cakes by replacing oil with inulin (Rodríguez‐García et al., 2014), and using alcoholic sugars as a sugar substitute in the formulation of low‐calorie cakes (Drabińska et al., 2018; Hao et al., 2016). Many researches have been conducted with the aim of producing functional cakes by partial replacing of the wheat flour in the cake formulation with different compounds such as *Elaeagnus angustifolia* flour (Emamifar et al., 2020; Zareh et al., 2016), flaxseed (Ayoubi, 2018), lentil flour (Khalil Zadeh et al., 2016), sorghum flour (Naghipour et al., 2013), and corn and rice flour mixture (Yanpi et al., 2018).

By increasing the percentage of jujube powder in the sponge cake formulation, the cohesiveness of the dough increased, but the hardness of sponge cake was reduced. Also the results showed that increasing the amount of *Elaeagnus angustifolia* flour in the cake formulation resulted in a decrease in the apparent viscosity of dough, fat, cake pH, volume, protein and increase in fiber content without any adverse effect on the physicochemical and sensory properties of the final product. This observation suggests that replacing wheat flour with *Elaeagnus angustifolia* flour as a functional raw material during cake preparation provides a suitable combination for cake formulation (Emamifar et al., 2020; Zareh et al., 2016).

Dhen et al. (2017) used AKF as a partial substitute for wheat flour to make dough. The results showed that the use of AKF as an alternative to wheat flour reduced the elasticity and consistency of the produced dough and showed thixotropic behavior (Dhen et al., 2017). Also Seker et al. (2010) showed that by the addition of AKF (10%, 20%, 30%, and 40%) to the cookie formulation, the tenderness of produced cookies reduced and the hardness increased. However, sensory evaluation showed that cookies containing AKF were accepted by panelists in all substitution percentages (Seker et al., 2010).

Apricot (*Prunus armeniaca*), a good source of nutrients, is one of the most familiar crops in the world. Apricot kernel is an important source of dietary protein as well as oil and fiber and also has high antioxidant and antimicrobial activity. In recent years, interest in the functional potential of byproducts has increased. Among them, apricot kernel as a byproduct of apricot fruit processing is known as a potential source of protein, oil, sugars, and fiber. Apricot kernels are mainly used in the production of oils, benzaldehydes, cosmetics, activated carbon, flavor, and flour. AKF is obtained from grinding the brain of the apricot kernels. It contains about 48% protein, mainly albumins; it also contains large amounts of potassium and magnesium, B vitamins, and tocopherol. Its oil is rich in polyunsaturated fatty acids (PUFA), especially oleic and linoleic. Considering these unique and special properties of AKF, the aim of this study was to investigate the effect of adding different ratios of AKF to cupcake formulations on the quality attributes of produced cupcake during the storage period.

## 2 MATERIAL AND METHODS

### 2.1 Raw materials

Fresh sweet apricot kernel was prepared from a factory located in Azarshahr, Iran. Wheat flour was purchased from a confectionery, and vanilla, baking powder, sugar, oil, eggs, and polyethylene bags from the local market. Eggs were purchased daily to preserve the freshness of the product.

### 2.2 Preparation of raw materials

In order to prepare AKF, the kernels were soaked in warm water for 30 min and their thin, brown brain shells were separated. The scalped kernels were then dried at ambient temperature for 4 days. To prevent possible oxidation that may occur during storage, AKF was prepared freshly one hour before cake production and added to the dough formulation in two ratios of 15% and 30% for the enrichment of cupcake.

### 2.3 Preparation of cake dough

Cake dough was prepared using the sugar‐dough method. The ratio of cake dough ingredients was selected based on the flour ratio. First, oil (57%) and sugar (72%) were mixed together and the dough was prepared with light cream color for 10 min. In the next step, the egg (72%) was added four or five times at intervals of 5–7 min, and after each step, it was well mixed. For aeration of flour, powdered materials, including baking powder (1.34%), powdered milk (2%), and vanilla (0.5%) were sifted three times with flour and added to the dough mixture. Finally, water (25%) was added and the mixing continued for about one minute (Peighambardoust, 2010). It must be noted that AKF with oil was added to the sugar and inserted into the cake dough. After dough preparation, its properties were evaluated.

### 2.4 Baking procedure

Forty‐five grams of prepared dough was poured into muffin molds immediately after mixing and baked in a baking oven (Alton, V202, Made in Iran) at 170°C for 25 min. After baking, the samples were cooled at ambient temperature for 30 min. The cakes were then packed in polyethylene packages and stored at room temperature until further experiments were performed.

### 2.5 Physical tests of dough

#### 2.5.1 Specific gravity of dough

The specific gravity of the cake dough is measured as a factor to check the amount of air bubbles entering the dough and the amount of air retention during dough mixing. The specific gravity of the dough was calculated by measuring the ratio of mass of dough to the mass of water used at 25°C using the same cup (Lin et al., 2017).

#### 2.5.2 Consistency of dough

To measure the consistency of cake dough, the dough was poured in a funnel with an inner diameter of 10 cm and a narrow inner diameter of 1.6 cm. The funnel was completely filled with dough, and then, the weight of the dough removed from it was measured for 15 s. The consistency of the dough was reported in grams of dough removed from the funnel per second. Larger numbers recorded indicate less consistency of dough (Pierce & Walker, 1987).

#### 2.5.3 Dough apparent viscosity

The apparent viscosity was measured by the Lakshminarayan et al. (2006) method using a Brookfield rotary viscometer (T2DV model, Made in America). In this way, the cake dough was transferred to a 600‐ml beaker, and the apparent viscosity was calculated at room temperature with spindle no. 5 and speed of 10 rpm after 1 min (Lakshminarayan et al., 2006).

### 2.6 Physical tests of cake

#### 2.6.1 Determining the baking loss of the cake

The percentage of cake baking loss was calculated from the difference in the weight of cake dough before baking and the cake samples after cooling and reaching the ambient temperature using Equation 1:

(1) $$\text{Baking loss}\left(\%\right)=\frac{W_{1}‐W_{2}}{W_{2}}\times 100$$

where *W*_1_ is the weight of dough (gr) and *W*_2_ is the weight of the cake (g).

#### 2.6.2 Measuring cake height

The height of the cake was measured using a digital caliper after production and cooling of the cake after one hour (Majzoobi et al., 2012).

#### 2.6.3 Measuring the volume of cake

Grain displacement method was used to measure the volume of different samples of cakes. In this method, after weighing an empty container, the volume of the container was measured with distilled water. Then, the container was completely filled using millet grains (the grain surface was completely flattened by a ruler drawn on the surface of the container), and after weighing, the weight and density of millet seeds was calculated from Equation 2:

(2) $$\text{Milletseed density}\left(\text{gr}/{\text{cm}}^{3}\right)=\frac{W_{2}‐W_{1}}{V}\times 100$$

where *W*_1_ is the weight of the empty container (g), *W*_2_ is the weight of the container along with millet seeds (g), and *V* is the volume of the container (cm^3^).

Then the cake is placed in the container and weighed (*W*_3_). Then the remaining empty space inside the container is filled and reweighed by millet grains completely and precisely to the surface of the container (*W*_4_). In this way, the weight of the added millet grains was obtained from the difference between these two values, and the volume of cakes was calculated using Equations 3 and 4 (Farahnaky & Majzoobi, 2008).

(3) $$VA=\frac{W_{4}‐W_{3}}{\text{millet}\;\text{grain}\;\text{density}}$$

where *W*_3_ is the weight of the container along with cake (g) and *W*_4_ is the weight of the container along with cake and millet grains (g).

(4) $$V_{B}=V‐V_{A}$$

where *V*_A_ and *V*_B_ are the volumes (cm^3^) of millet grains and final cake, respectively.

#### 2.6.4 Cake bulk density

The bulk density was calculated by measuring the mass‐to‐volume ratio of cake.

#### 2.6.5 Determination of cake moisture

Five grams of cake crust and crumb was separated and completely mixed together, and its moisture content was measured by the drying method in a hot oven (105°C) until it reached a constant weight according to the AACC standard method no. 15‐44 (AACC, 1999).

#### 2.6.6 Cake symmetry

Cake symmetry was measured using the AACC‐1091 method using a transparent ruler (AACC, 1999).

#### 2.6.7 Evaluation of hardness of cake texture

Hardness was considered as the maximum resistance to deformation imported by 40% compression in tissue. For this purpose, the hardness of cake samples was measured using Sentam tissue analysis (MODEL 5 STM, Made in Iran) according to the Hess and Setser (1983) method with slight modifications. To this end, a cubic piece of 2.54‐cm dimension was separated from the crumb of the cake without crust, and the probe compressed 1 cm (40%) of the tissue. The force of the device loading cell was 5.7 N, and the probe speed was 50 mm/min. The compressive force of the sample was reported in Newton's terms. Higher force entering the samples indicated greater hardness of the samples (Hess & Setser, 1983).

#### 2.6.8 Cake color evaluation

The image processing method was used to investigate the cake's color characteristics. In order to evaluate the color and determine the color factors (L*, a*, b*), for the crust (surface of complete samples) and for the crumb, a slice of cake was prepared. Samples were placed in a cardboard box with dimensions of 20 × 20×30 for photography. The distance between the samples from the camera (Samsung Camera Model 5A) was 30 cm. The obtained photos were examined by Photoshop software, and the L*, a*, and b* factors were determined for each point (Aghajani et al., 2018; Gohari Ardabili et al., 2021; Hashemi Shahraki et al., 2014).

### 2.7 Sensory evaluation

Five‐point hedonic sensory tests were used to evaluate different cake treatments for sensory properties. For this purpose, six evaluators were selected from the staff and students of the faculty and trained to understand the concepts of cake pores, softness and hardness of the texture, crust and crumb color of the cake, flavor, and overall acceptance of cake based on Lee et al. (2008), and each coded sample was given to the panelists. In this evaluation, the highest score of 5 (very good) and the lowest score of 1 (very bad) were considered as the range of points earned (Daraei Garmakhany et al., 2011; Lee et al., 2008). In order to investigate the effect of storage time factor on texture and quality of the product, sensory evaluation was performed on days 0, 7, and 14 after baking. The sensory evaluation test was done with two replications.

### 2.8 Statistical analysis and experimental design

The effect of adding AKF and storage time on the qualitative properties of dough and cake product was investigated as factorial in a completely randomized design (CRD) and analyzed by SAS software (9.3.1). The mean of treatments was compared using Duncan's multiple range test at the 95% confidence level. Excel software (2007) was used to draw diagrams. All the experiments were conducted in three replications. The optimization of AKF‐enriched cake production was done by using Design Expert software version 11.1.2.0. (Stat‐Ease Inc., Minneapolis, MN, USA). For experiment designing, the central composite design (CCD) was used. Independent variables, including AKF concentration (X_1_) and storage time (X_2_) at three different coded levels, low (−1), medium (0), and high (+1), were investigated. The ranges of independent variables were determined from preliminary experiments. The range of AKF concentration and storage time were 0%–30% and 0–14 days, respectively (Table 1).

**TABLE 1:** Independent variables and their applied levels for optimizing physicochemical properties of apricot kernel flour (AKF)‐enriched cupcake

Independent variables	Variable level		
	−1	0	+1
Storage period (days)	0	7	14
AKF concentration (%)	0	15	30

### 2.9 Optimization

For finding the optimal condition for AKF‐enriched cupcake production, independent variable, i.e. AKF concentration puts up in the chosen range, and storage time was chosen maximum and responses such as cake total acceptance, flavor and texture score, height (mm), symmetry (mm), and volume (cm^3^) considered as maximum while baking loss (%) and instrumental hardness were considered minimum other quality parameters were chosen at range of obtained results during the experiments. The optimization process and variables constrains are presented in Table 2.

**TABLE 2:** The optimal condition and constrain for variables and their applied levels for optimizing physicochemical properties of apricot kernel flour (AKF)‐enriched cupcake

Variables	Goal	Lower	Upper	Lower	Upper	Importance
A:apricot flour concentration	Is in the range	0	30	1	1	3
B:storage time	Maximize	0	14	1	1	3
Apparent viscosity (cP)	Is in the range	31,740	71,502	1	1	3
Falling rate	Is in the range	1.3667	3.5867	1	1	3
Specific gravity	None	1.046460954	1.08667	1	1	3
Total acceptance	Maximize	3.3333	4.25	1	1	3
Flavor score	Maximize	3.25	4.1667	1	1	3
Porosity score	None	3.5833	4.75	1	1	3
Texture score	Maximize	2.6667	4.75	1	1	3
Crumb color	None	3.75	4.6667	1	1	3
Crust color	None	3	4.6667	1	1	3
Height (mm)	Maximize	37.23	44.1	1	1	3
Symmetry (mm)	Maximize	−0.1	0.3	1	1	3
Volume (cm^3^)	Maximize	60.425	84.70167	1	1	3
Bulk density (g/cm^3^)	Is in the range	0.454867069	0.665	1	1	3
Baking loss (%)	Minimize	10.255	14.38222	1	1	3
Hardness (*N*)	Minimize	1.555	35.41	1	1	3
Moisture content (%)	Is in the range	14.72666667	19.37	1	1	3

## 3 RESULTS AND DISCUSSION

### 3.1 Effect of AKF addition on the properties of cupcake dough

#### 3.1.1 Special gravity of cake dough

**FIGURE 1:**
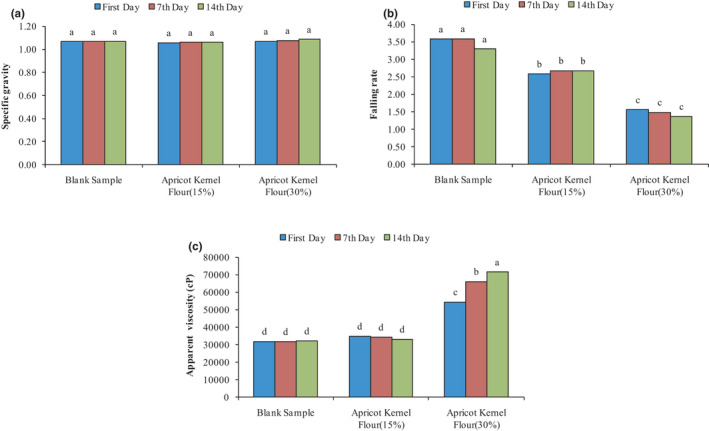
Changes in the specific gravity (a), falling rate (b), and apparent viscosity (c) of cake batter under the effect of AKF addition during the storage period

The results of the effect of AKF addition, on the specific gravity of cupcake dough, are presented in Figure 1A. As can be seen, adding AKF to the cupcake dough did not cause significant changes in the specific gravity of the dough. The dough specific gravity of the control sample had no significant difference with samples containing 15% and 30% AKF (*p* > .05), in agreement with the results of the Shokri Busjin Study (2004). He used tragacanth gum as a substitute for fat in cake and observed that the uniformity and specific gravity of the cake dough were not affected by the percentage of tragacanth gum and oil (Shokri Busjin, 2004).

Huang and Yang (2019) investigated the effect of Eucheuma powder, which contains a high amount of dietary fiber (69.33%), in order to replace 0%, 5%, 10%, 15%, and 20% of wheat flour to make sponge cakes, referred to as the control, EP5, EP10, EP15, and EP20, respectively. The results revealed that replacing flour with Eucheuma powder caused an increase in the specific gravity of the cake batters when added to the batter from 0.505 (control sample) to 0.559 (EP20 sample). This clearly revealed that Eucheuma powder had better ability to prevent the foaming capabilities of eggs than wheat flour. Eucheuma had higher WHC than that of cake flour (0.64 g of water/g of powder). This property may change the influence of water with proteins, starch, and sugar, which might cause the decrease in foaming capabilities of eggs. Meanwhile, the oil absorption capacity (OAC) of Eucheuma powder was also very high (the OAC of cake flour was 0.61 g of oil/g of powder), suggesting it is helpful for the stability of the batter system. Therefore, the batters with Eucheuma powder were not damaged totally.

Lin et al. (2017) showed that the substitution of eggs by SPI led to a significant increase in the specific gravity of the batter (1.03 vs. 1.13) and a decrease in the specific volume with respect to the control (2.08 vs. 1.47 cm/g). Therefore, the results suggest that substitution with SPI cannot guarantee the specific gravity of the batter and the specific volume of the cakes. However, the presence of baking additives, such as xanthan gum, mono and diglycerides, and soy lecithin, reduced the specific gravity of the batter significantly (*p* < .05) compared with the eggless cake batters (SPI only).

#### 3.1.2 Consistency of cake dough

The consistency of the cake dough is important in maintaining the physical air that was originally created in the dough during the mixing process and determines the speed at which the air bubbles rise toward the surface of the cake. In cake batters with too low and too high consistency, due to the higher speed of moving air bubbles toward the surface and the slow expansion, respectively, the specific volumes and height of cakes are affected (Aydogdu et al., 2018). In these doughs, the crust is formed before the inner parts of the cake, and this crust prevents the air bubbles moving toward the surface (Frye & Setser, 1992). In contrast, in dough with high consistency, while not forming enough air bubbles during stirring the dough, the dough expansion during baking is prevented (Gomez et al., 2010). It is advisable to consider the consistency of cake dough high enough to maintain its pumping and molding capability, so that it can maintain more air bubbles during the mechanical phase of mixing or mechanical aeration in the dough (Peighambardoust, 2010).

The effect of adding AKF on the consistency of cake dough loss is presented in Figure 1B. As can be seen over 14 days, the consistency of the blank sample dough with the cake dough containing 15% and 30% AKF differed significantly (*p* < .05), and the addition of AKF to cake dough increased the consistency of the dough so that the control sample had the highest flow rate and the lowest consistency, and dough containing 30% AKF had the lowest flow rate and maximum consistency. It seems that the fiber and proteins available in AKF due to the ability of water absorption and reaction with starch and flour proteins trap the amount of free water and formed the network in the dough that is effective in the movement of dough particles and thus increase the consistency of the dough. The results were in agreement with the research conducted by Esmail Pour (2012), which investigated the effect of adding date kernel powder on the quality characteristics of sponge cake and observed that the addition of date kernel powder increased the consistency of cake dough, which can be due to the high fiber in date kernel powder (Esmail Pour, 2012). Also, Gomez et al. (2010), investigating the effect of fiber type (wheat bran, oat bran, and microcrystalline cellulose), particle size, and replacement percentage on dough and cake properties, showed that with the increasing bran percentage, dough consistency index increased, flow index decreased, and cake volume increased (Gomez et al., 2010).

#### 3.1.3 Apparent viscosity of cake dough

Apparent viscosity of dough is one of the most important physical properties in baking cakes and due to maintain air bubbles and produced gases in the dough, it increases the stability of the dough and is considered as the control factor for the final volume of cake. If the dough is not viscose enough, it connects the gas bubbles to each other, and as a result, large bubbles come to the surface and exit the dough system (Ashwini et al., 2009). The results of the effect of AKF addition on the dough apparent viscosity are presented in Figure 1C. As can be seen by the addition of AKF to the cake formulation, the apparent viscosity of the dough increased so that the dough containing 30% AKF on the 14th day had the highest apparent viscosity. The apparent viscosity of the dough of the control sample and dough containing 15% AKF for 14 days was not significantly different (*p* > .05), while the cake dough containing 30% AKF was significantly different from both of them (control and sample containing 15% AKF) (*p* > .05). The minimum apparent viscosity was related to the control and sample containing 15% AKF during the 14‐day storage period. The apparent viscosity of cake dough containing 30% AKF increased significantly for 14 days (*p* < .05). It seems that the fiber and proteins present in AKF have the ability to absorb water and react with starch and flour proteins, by formation of the network, trap an amount of free water that is effective in the movement of dough particles, and therefore increase the apparent viscosity of the dough, which is in accordance with the results of other researchers (Dadkhah et al., 2012; Salehi et al., 2016).

Lin et al. (2017) showed that the viscosity of the eggless cake batters containing soy protein isolate (SPI) was higher than the control batter samples (*p* < .05). They also showed that xanthan gum (XN) addition also increased batter viscosity that may be related to the high water‐binding capacity of SPI and XN, while the emulsifiers decreased it. However, combinations of XN and emulsifiers lead to a reduction in cake batter viscosity that may be caused by the delay of starch gelatinisation due to the presence of emulsifiers.

Huang and Yang (2019) showed that all batter samples containing Eucheuma powder had higher viscosity than the control sample at the same shear rate, which may be due to the high amount of carrageenan in the Eucheuma powder. Also, Huang et al. (2021) showed that increasing the κ‐carrageenan concentrations led to an increase in the viscosity of the sample, which may be due to the entanglement of κ‐carrageenan with starch and gluten.

Jahanbakhshi and Ansari (2020) showed that batter viscosity and consistency decreased (from 27,430 to 11,030 centipoise and from 8.67 to 13.42 g/s respectively) with increased amounts of olive stone powder (OSP).

### 3.2 The effect of AKF addition on the physical and apparent properties of cupcake

#### 3.2.1 Baking loss of cupcake

**FIGURE 2:**
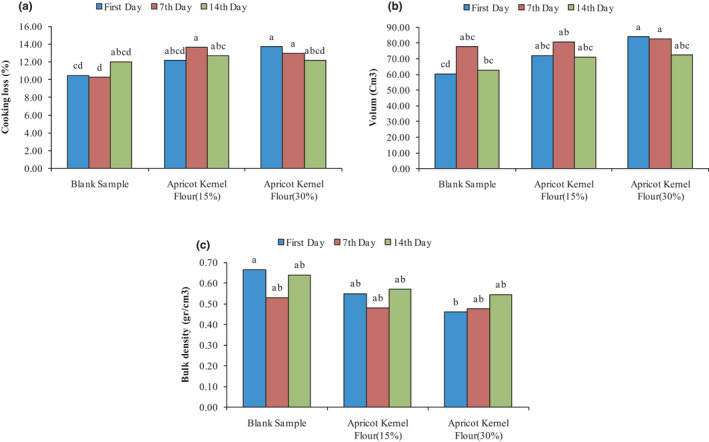
Changes in the cooking loss (a), volume (b), and bulk density (c) of produced cake under the effect of AKF addition during the storage period

During the baking process, due to the penetration of heat into the dough texture, liquid expansion occurs and internal steam pressure increases. Therefore, weight loss as a baking loss occurs due to the release of internal gases and degradation. The baking loss changes the structure and reduces the shelf life of of the cake. The results of AKF addition on cake baking loss are presented in Figure 2A. As can be seen on day 0, there was a significant difference between the control sample and the sample containing 30% AKF, and also, on the seventh day of the storage period, there was a significant difference between the samples containing 15 and 30% AKF with the control sample (*p* < .05). On the 14th day of the storage period, control samples and samples containing 15% and 30% AKF were not significantly different from each other (*p* > .05). The results were in accordance with the research conducted by Abdolnabipour et al. (2019) who used sesame meal flour and soy water‐soluble polysaccharide to improve the quality of the cake and showed that increasing the percentage of sesame meal flour significantly increased the weight loss of the cake. The reason for the increase in cake baking loss is the inability of sesame meal to hold water, which increases the loss of water during the baking process (Abdolnabipour et al., 2019).

#### 3.2.2 Cake volume

The volume of cakes indicates the amount of carbon dioxide and ammonia gas produced by the addition of chemical leavening agents used in the dough formula and the range of changes in the cake crumb during baking. Absorbent compounds and additives participating in the cake baking process determine this feature. Jahanbakhshi and Ansari (2020) showed that with increased amounts of olive stone powder (OSP), the specific volume of the cakes increased (from 2.08 to 6.21 cm^3^/g). Noorlaila et al. (2020) reported that hydrocolloid cakes (containing both XG and HPMC) had lower volume compared with control cake (*p* < .05).

Figure 2B shows the results of the addition of AKF on cake volume during the 14‐day storage period. As can be seen, the addition of AKF has increased the cake volume. Only on day 0 of the storage period was there a significant difference between the control samples and samples containing 15% and 30% AKF (*p* < .05) in terms of cake volume. On days 7 and 14, there was no significant difference between control samples and samples containing 15% and 30% AKF. In general, the control sample had the lowest cake volume on day 0, and the samples containing 30% of AKF had the highest cake volume on day 0. The results were in agreement with research conducted by other researchers (Abdolnabipour et al., 2019; Dadkhah et al., 2012).

Lin et al. (2017) reported that the addition of 1% of any emulsifier, or in combination with XN, increased the specific volumes of the SPI cakes to a level similar to that of the control cake. The cake volume is a result of the stabilizing proteins that form a strong thermal gel that ensures cake structure; therefore, high volume shows the presence of proteins with good gel‐forming properties, such as eggs.

#### 3.2.3 Cake bulk density

In general, dough density is associated with the amount of air bubbles present (Gomez et al., 2010). There is an inverse relationship between the bulk density and the volume of the cake; the higher volume of the cake results in the cake with lower bulk density. The results of the bulk density are presented in Figure 2C. According to the results, cakes with high percentages of AKF had the highest volume, so naturally, they had the lowest bulk density. The results showed that only on day 0 of the storage period, the addition of AKF to the cupcake dough caused a significant decrease in bulk density (*p* < .05), so that the control sample had the highest bulk density and treatment containing 30% AKF had the lowest density. On days 7 and 14 of the storage period, the cupcake density of samples containing 15% and 30% of AKF was not significantly different (*p* > .05). Sanchez‐Pardo et al. (2010) added a product combination beta‐glucan oat with dextrin and modified starch to the cake formula and observed that the volume of produced cakes increased and their density decreased (Sanchez‐Pardo et al., 2010). Salehi et al. (2016) showed that with the increasing apple powder percentage, the volume of cakes decreased significantly and the density increased (Salehi et al., 2016).

#### 3.2.4 Moisture content of cake

**FIGURE 3:**
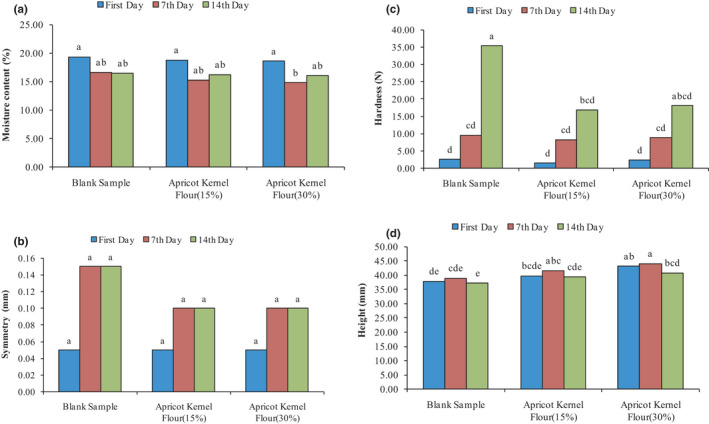
Changes in the moisture content (a), hardness (b), symmetry (c), and height (d) of produced cake under the effect of AKF addition during the storage period

In Figure 3A, the effect of AKF addition on the moisture content of cupcakes during 14 days of storage period is presented. As can be seen in a total of 14 days, the moisture content of the control sample was not significantly different from the moisture content of the cakes containing 15% and 30% AKF (*p* > .05). Evaluation of the effect of storage day shows that, over time, the moisture content of cake samples decreased (*p* > .05).

Jahanbakhshi and Ansari (2020) showed that adding 15% OSP and more of it reduced the moisture contents of the cakes significantly, but no significant difference was observed between the control and the 15% OSP containing samples in terms of moisture content. This may be due to the poor water absorption and water retention ability of OSP, resulting in a greater moisture loss during the baking process.

#### 3.2.5 Hardness of cake texture

Texture is a very important quality characteristic in bakery products and can determine the duration of product storage. This indicates that during the storage period, even under conditions that prevent moisture loss, cake may lose its freshness, especially at 15–20°C, which is probably due to starch retrogradation, the reaction between starch protein, as well as water migration. Different factors such as the amount of fat, sugar, and liquid are effective on cake tissue. The effect of adding AKF and the effect of storage time on the hardness of cupcake during the 14‐day storage period is presented in Figure 3B. As can be seen, until the seventh day of storage period, the addition of different percentages of AKF had no significant effect on the hardness of cupcake (*p* > .05). The highest amount of cupcake hardness was related to the control sample on the 14th day of storage while samples containing 15% AKF on the 14th day of storage showed the lowest hardness. Evaluation of the effect of storage day on the hardness of cupcake showed that the storage day had no significant effect on hardness, and over time, the softness of the cupcake decreased (*p* > .05). The available fiber in AKF has strong water absorption properties and can provide the same function as fat, so the texture of control samples and samples containing AKF is not statistically different (*p* > .05). The soft texture of the cupcake sample containing 15 and 30% AKF on the 14th day of storage period can be attributed to the presence of a significant amount of fiber and fat in AKF, which is in agreement with the results of other researchers. Dadkhah et al. (2012) stated that the cake containing oat bran at the replacement level of 20% shortening was not statistically different from the control cake in terms of hardness. Jahanbakhshi and Ansari (2020) revealed a significant decrease in cake hardness as the amount of OSP increased in formulation. In general, control cake sample with low specific volume showed a harder texture.

#### 3.2.6 Cake symmetry

The results of the symmetry test are shown in Figure 3C. As can be seen, the addition of AKF to cake dough did not cause changes in the cake symmetry index, and the symmetry of the control sample with samples containing 15 and 30% AKF on days 0, 7, and 14 of the storage period did not significantly differ (*p* > .05). Therefore, the use of AKF did not have any negative effect on the symmetry of the cake. Gómez et al. (2007) reported that low‐calorie cake containing pectin had the same symmetry index as the control sample. In general, layer cakes with higher volumes indicate higher height and symmetry (this last feature is mainly related to the type of used baking powder), but cake containing pectin, despite larger volume than the control sample, had the same height and symmetry as the control sample (Gómez et al., 2007).

#### 3.2.7 Cake height

The effect of adding AKF to cake height in Figure 3D is presented. Over the 14‐day storage period, the height of the control sample was significantly different from the samples containing 30% AKF, and the addition of AKF increased the cake height (*p* < .05). The control sample had the lowest height on the 14th day of the storage period, and the cake containing 30% AKF had the highest height on the seventh day of the storage period. Perhaps the main reason for the increase in cake height is the presence of chemical gas–producing compounds such as baking powder, operation of kneading and mixing of air in cake dough, and finally evaporation of water molecules during baking. These factors also have a direct effect on cake volume increase.

### 3.3 Effect of adding AKF on the crust and crumb color properties of cupcake

**FIGURE 4:**
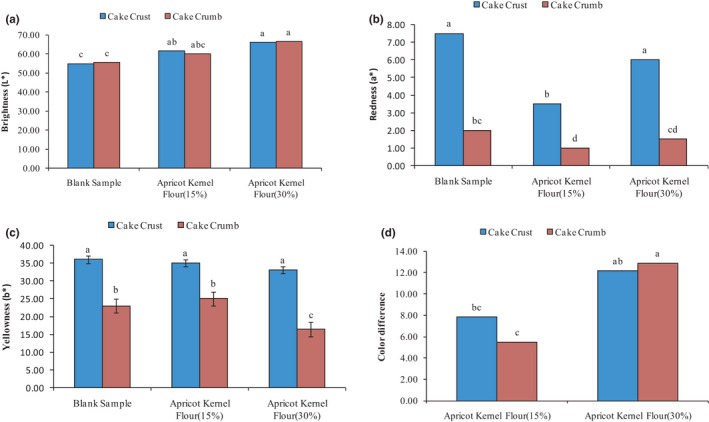
Changes in color indexes: A‐brightness (L*), B‐redness (a*), C‐yellowness (b*), and D‐color difference of produced cake under the effect of AKF addition during the storage period

Color is one of the most important indicators of food that can have a significant impact on consumers' attention. Differences in cake formulations can affect their color. The results of color analysis of cupcakes containing different percentages of AKF showed that the increase in AKF significantly increased the amount of L* (brightness) of the cake crust and crumb, and the component a* (redness) of the samples containing 15% AKF significantly decreased (0.05 < *p*). The amount of b* component (yellowness) of the crust and crumb of the control cake and samples containing 15% and 30% AKF did not change significantly; only the amount of yellowness of the cake crumb in samples containing 30% AKF significantly decreased (Figure 4). As a result, the cakes containing 15% and 30% AKF were brighter than the control sample.

As it turns out, the color of the crust is influenced by Millard's browning reactions. Since fibers cannot affect the amount of sugars and amino acids due to their chemical nature, the lack of significant effects of fibers on the color of cake samples can be justified by such an argument. In Figure 4D, the effect of adding AKF on the cake color difference index is shown.

As can be seen, the addition of AKF to the cake dough caused a significant difference in the L*, a*, and b* components of the cake crust and crumb of the control sample with samples containing 15% and 30% AKF (*p* < .05). The cake sample containing 30% AKF has the highest crust and crumb color difference (Δ*E*) compared with the control sample and sample containing 15% AKF. Salehi (2018) showed increasing the percentage of balangu seed gum increased the amount of lightness (L*). Increasing the cake's lightness by increasing the percentage of gum is due to the increase in the cake's volume, which makes the internal texture of the cakes brighter. There was no significant difference between the samples in terms of a* index, but b* decreased with increasing gum concentration (Salehi, 2018).

### 3.4 Effect of adding AKF on the sensory properties of cupcake

#### 3.4.1 Sensory evaluation of the crust and crumb color of cakes

**FIGURE 5:**
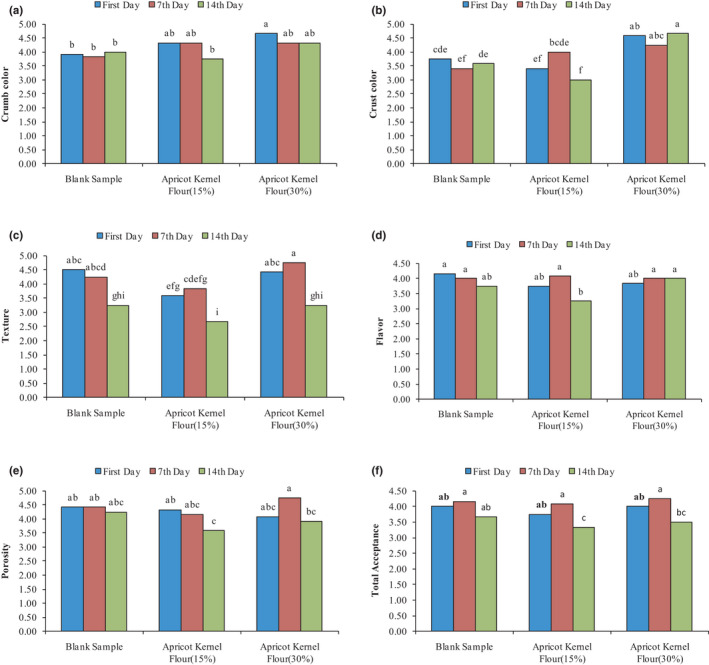
The effect of AKF addition on sensory properties variation (a, crumb color, b, crust color, c, texture, d, flavor, e, porosity, f, total acceptance) of produced cake during the storage period

Figure 5A1, A2 shows the sensory evaluation results of the crumb and crust color of cake samples containing AKF. As can be seen, only on day 0, the crumb color score of the cake samples containing 30% AKF had a significant increase compared with the control sample (*p* < .05), and on the other days of the storage period, the control samples and the cake samples containing 15% and 30% AKF were not different (*p* > .05). On days 0 and 7 of the storage period, there was a significant difference between cake samples containing 30% AKF with the control sample in terms of crust color score, but on the 14th day of the storage period, the significant difference between control samples and cake samples containing 15 and 30% AKF was observed (*p* < .05). The addition of AKF to the cake dough increased and improved the cake color index, so that the cake samples containing 30% AKF on day 14 had the highest crust color score, and on day 0, they had the highest crumb color score (*p* < .05).

#### 3.4.2 Sensory evaluation of the crust and crumb texture of cakes

Since the texture of foods affects consumer acceptance, it is an important criterion of food quality and refers to a group of physical characteristics of foods, which is felt and understood by touching, which is generally done in the mouth and through the palate and tongue. The results of crumb and crust texture sensory evaluation of cake samples containing AKF showed a significant decrease in the texture score of cake samples containing 15% AKF compared with the control sample, and only on day 0 of the storage period, this decrease was significant (*p* < .05). AKF containing samples and control sample did not differ significantly from with each other during the 14‐day storage period (*p* > .05). The highest score belonged to the cake samples containing 30% AKF on the seventh day, and the lowest score was related to cake samples containing 15% AKF on the 14th day of the storage period (Figure 5C).

#### 3.4.3 Cake flavor

The results of flavor analysis showed that during the storage period (14 days), the flavor index of the control sample with cake containing 15% and 30% AKF did not differ significantly from each other (*p* > .05). The lowest sensory score of flavor was related to the cake samples containing 15% AKF on day 14, and the highest score was related to control cake on day 0 of the storage (Figure 5D).

#### 3.4.4 Porosity of cake

The results of AKF addition on the cake porosity during the 14‐days storage period are presented in Figure 5E. The results showed that the porosity index of the control sample, and the samples containing 15% and 30% AKF were not significantly different from each other (*p* > .05). Cake samples containing 30% AKF on the seventh day of storage had the highest porosity score while cake samples containing 15% AKF on day 14 had the lowest porosity score (*p* > .05).

#### 3.4.5 Overall acceptance

The results of the addition of AKF on the overall acceptance index during 14 days of storage are presented in Figure 5F. As can be seen, cake samples containing AKF on the 14th day had a significant difference in the overall acceptance index (*p* < .05). The lowest overall acceptance score was related to the cake samples containing 15% AKF on the 14th day of storage. The results of sensory evaluation showed that the addition of 30% AKF had a significant effect on the sensory properties of crust and crumb color, texture, porosity, flavor, and overall acceptance compared with the control sample and improved these quality attributes. Cake samples containing 30% AKF were identified as the best samples by panelists.

### 3.5 Response surface optimization results

**FIGURE 6:**
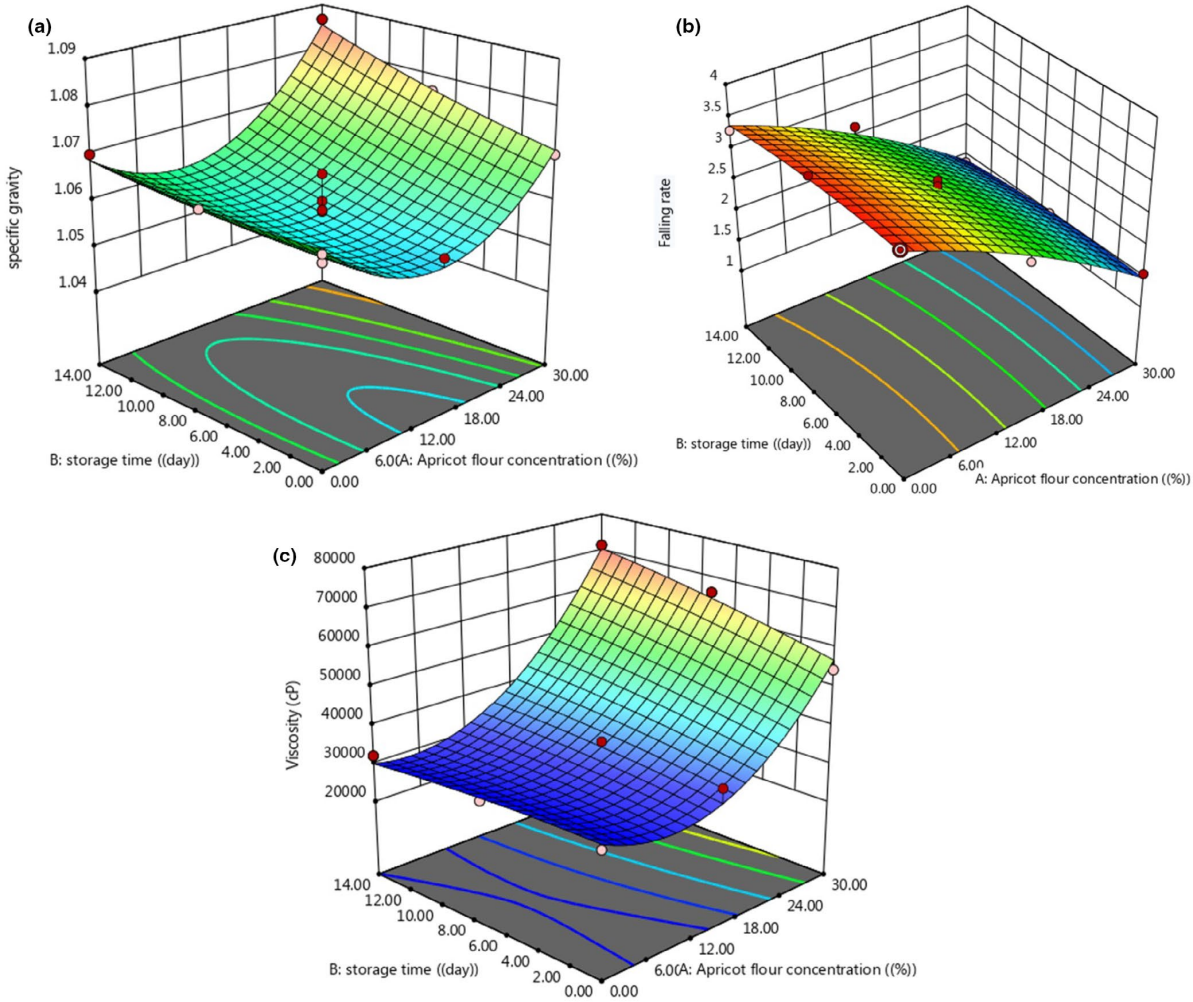
3D surface plot of variation of the specific gravity (a), falling rate (b), and apparent viscosity (c) of cake dough under the effect of AKF addition during the storage period

**FIGURE 7:**
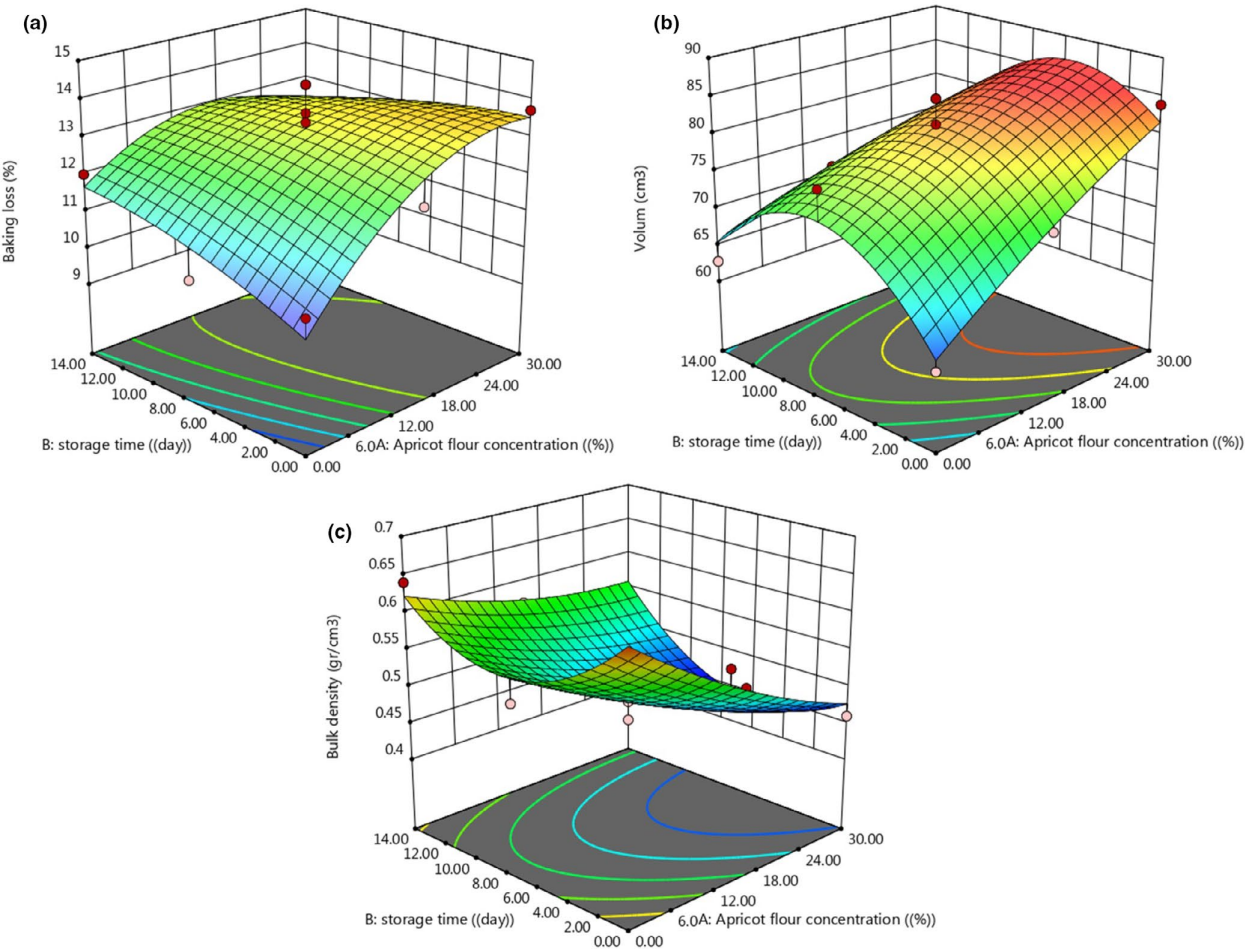
3D surface plot of the variation of the baking loss (a), volume (b), and bulk density (c) of produced cake under the effect of AKF addition during the storage period

**FIGURE 8:**
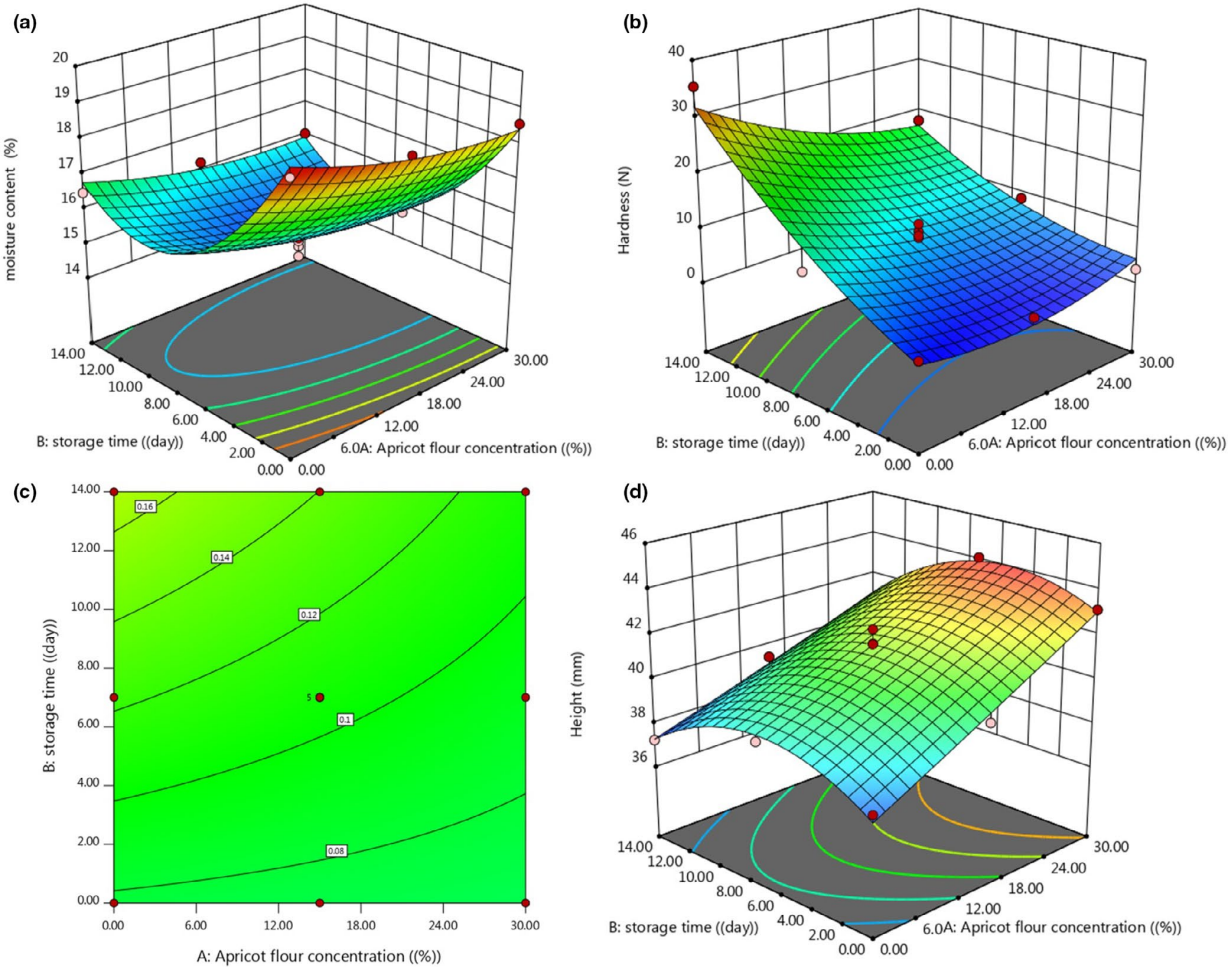
3D surface and contour plot of the variation in the moisture content (a), hardness (b), symmetry (c), and height (d) of produced cake under the effect of AKF addition during the storage period

**FIGURE 9:**
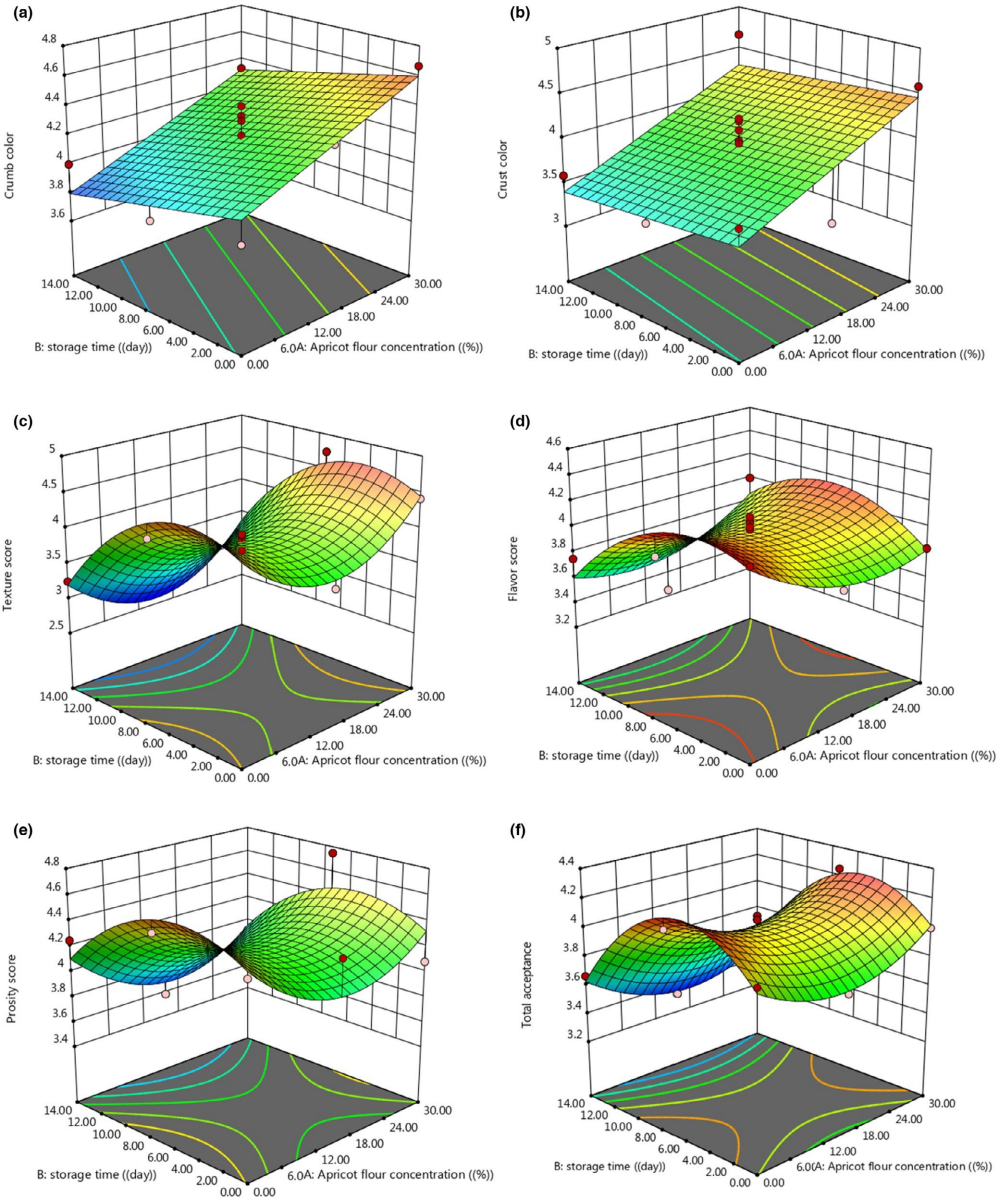
3D surface plot of the effect of AKF addition on sensory property variation (a, crumb color; b, crust color; c, texture; d, flavor; e, porosity; f, total acceptance) of produced cupcake during the storage period

The independent variables and their applied levels for optimizing physicochemical properties of apricot kernel flour (AKF)‐enriched cupcake are shown in Table 1. Thirteen experiments were done according to design with two factors and three levels for each variable. Different quantitative responses represent that the quality of the cupcake was considered for optimization. The analysis of variance (ANOVA) and lack of fit test were considered for the significance of models of regression, and 3D surface and contour plot of the significant models are shown as Figures 6, 7, 8, 9.

Figure 6 shows the 3D surfaces of the effect of AKF concentration and storage period on different quality attributes of produced cupcake dough. As can be seen in Figure 6, variation of specific gravity, consistency and apparent viscosity of cake dough under the effect of AKF concentration and storage period can be predicted by quadratic (second order) models. The specific gravity, falling rate, and apparent viscosity increased by increase in storage time. Increase in AKF to the cupcake dough increased the specific gravity and apparent viscosity and decreased the falling rate (increased consistency) significantly. As shown in Figure 6, the AKF addition is more effective on the specific gravity of the dough, consistency, and apparent viscosity compared with the storage period. This may be due to the role of fiber and proteins that exist in AKF, which can form a network by absorbing water and react with starch and flour proteins. By forming this type of network, free water content and the movement of dough particles were affected; therefore, the apparent viscosity and consistency of the dough increased, which is in accordance with the results of other researchers (Dadkhah et al., 2012; Salehi et al., 2016).

The 3D surface plot of the variation of the baking loss, volume, and bulk density of produced cupcake under the effect of AKF addition during the storage period is illustrated in Figure 7. Baking loss, cake volume, and cake bulk density are important factors used to verify the quality of the cupcake and variation of these parameters under the effect of AKF addition and the storage period can be predicted by quadratic (second‐order) models. Backing loss increased with an increase in storage time and AKF concentration. At higher AKF concentrations (more than 24) by increasing storage time, baking loss decreased that may be an interaction of fiber and protein content of AKF with flour, starch, and protein, and prevention of water migration forms the crumb to the crust of the cake. As can be seen, the AKF concentration has more effect on baking loss compared with the storage period (*p* < .05).

Bulk density and cake volume have the reverse trend with each other, and any factor that increases volume decreases bulk density and so any factor that decreases volume increases bulk density. As shown in Figure 7, cake volume and bulk density was increased and decreased, respectively, by increasing AKF concentration to the cake formulation. With increase of storage time to 10 days, the cake volume increased while bulk density decreased. After 10 days of the storage period, the reverse trend was observed in terms of cake volume and bulk density. The AKF concentration affects cake volume and bulk density more than storage period.

The 3D surface and contour plot of the variation of the moisture content, hardness, symmetry, and height of produced cupcake as a function of AKF addition during the storage period are illustrated in Figure 8. As can be seen from Figure 8, quadratic (second‐order) models were used to predict the variation of these parameters under the effect of AKF addition and storage time. The storage period has a significant effect on cupcake moisture content, hardness, symmetry, and height (*p* < .05). By increasing storage time, cake hardness and symmetry increased, but the moisture content decreased. The cake height increased over time (10 days) and then decreased with an increase in storage time. An AKF addition to the cake formulation leads to an increase or preserves the moisture content, hardness, symmetry, and the height of cake compared with the control sample.

The 3D surface plots of AKF addition effect on sensory properties variation (A‐crumb color, B‐crust color, C‐texture, D‐flavor, E‐porosity, F‐total acceptance) of final cupcake during the storage period are illustrated in Figure 9. As can be seen from Figure 9, linear (first‐order) model was used to predict the variation of crust and crumb color score, and quadratic (second‐order) models were applied to predict the variation of other sensory attributes of cupcake under the effect of AKF addition and storage time. Figure 9 shows that the addition of AKF cake formulation has a significant effect on cupcake crumb and crust color sensory score, and with an increase in AKF concentration, crumb and crust color score increased linearly (*p* < .05). As can be seen over the storage time, the crust and crumb color score decreased (*p* > .05). The texture, flavor, porosity, and total acceptance sensory score show a similar trend, and all these properties increased with increasing storage time (till 10 days of storage period) but decreased from 10 to 14 days of storage. An increase in AKF concentration to 18% leads to a decrease in the texture, flavor, porosity, and total acceptance sensory score; by adding higher concentrations (18%–30% AKF), the sensory score of these parameters increased.

### 3.6 Optimized conditions

**FIGURE 10:**
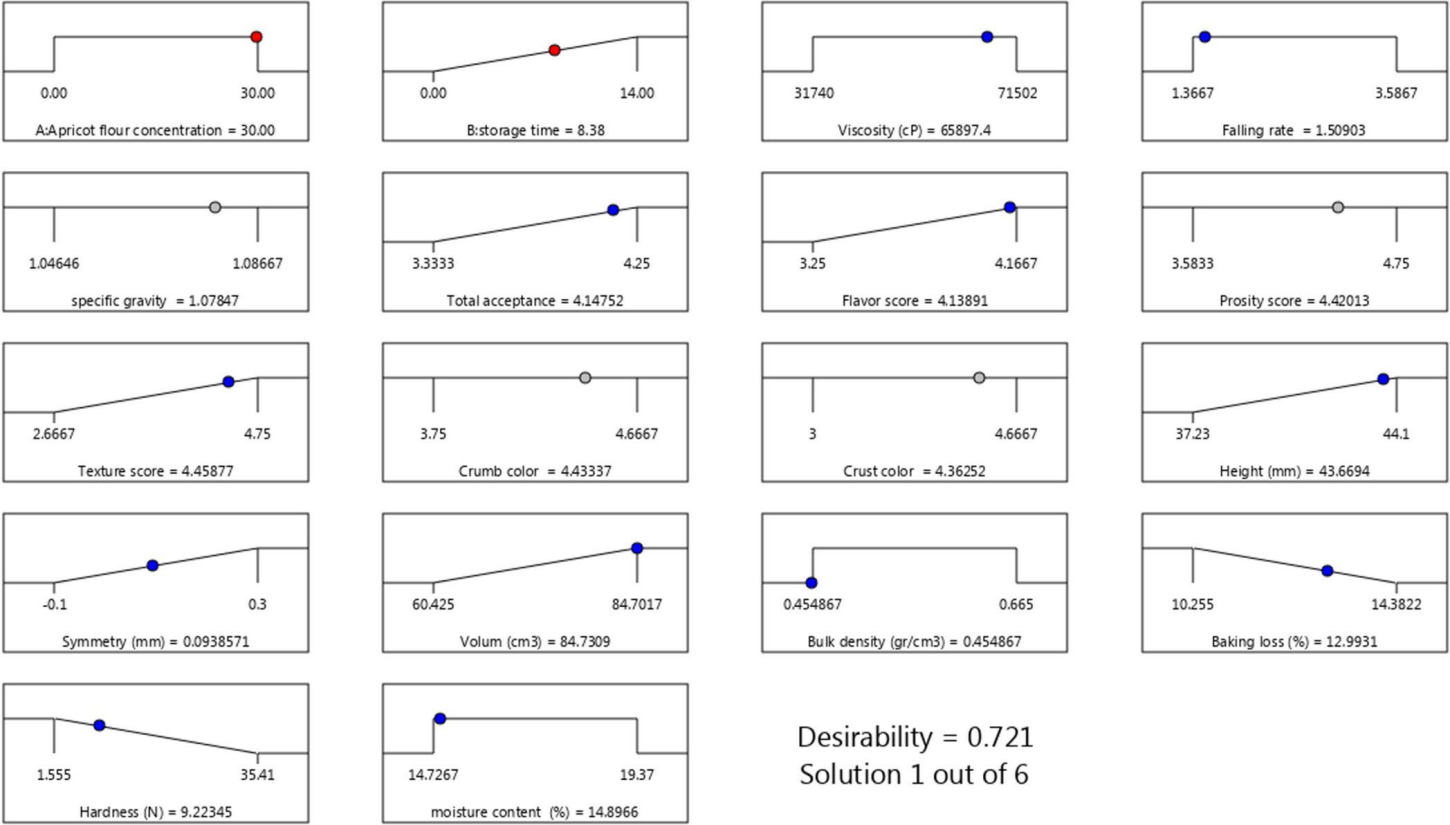
The optimum conditions for producing AKF‐enriched cupcake under the effect of different concentrations of AKF and storage period

Response surface method with CCD was used to find out the optimum conditions for producing AKF‐enriched cupcake. Thirteen experiments were analyzed, according to the design, with two independent factors at three levels for each variable. The results for the different cupcake samples showed that the optimum levels of AKF concentration and storage period as independent variables were 30% and 8.38 days, respectively. The optimal levels for the dough apparent viscosity, consistency, and specific gravity were 65,897.4 cP, 1.509, and 1.0785 gr/cm^3^, respectively. Under these optimum conditions, the sensory and quality attributes of produced cupcake including total acceptance, flavor, porosity, texture, crumb and crust color score, height, symmetry, volume, bulk density, baking loss, hardness, and moisture content were 4.148, 4.139, 4.42, 4.459, 4.433, 4.363, 43.669 (mm), 0.094 (mm), 84.731 gr/cm^3^, 12.993%, 9.223 N and 14.897%, respectively, with a desirability of 72.1% are shown in Figure 10.

## 4 CONCLUSION

Cake is one of the foods that peoples all over the world love to eat but due to the presence of sugar and oil can be classified in the category of high‐calorie foods. Nowadays, people's demand for high‐quality, functional foods and lower calorie content has increased in order to promote health and prevent problems such as obesity and diabetes. Changes in food formulations (reduction in sugar and oil and the use of their alternatives) cause structural and rheological changes in cake and cake dough. These changes may produce different results depending on the types of applied compounds and the different formulations produced. The aim of this study was to investigate the effect of adding different levels of AKF on physicochemical and sensory properties of cake samples in order to determine the optimum formulation. Sensory evaluations showed that the addition of 30% AKF improved the sensory attributes of crust and crumb color, texture, porosity, flavor, and overall acceptability compared with the control sample, and cake samples containing 30% AKF were recognized as the best samples by panelists. The results of optimization showed that by increasing the satisfactory amount of AKF (30%), a functional product with high nutritional value can be prepared.

## CONFLICT OF INTEREST

The authors declare that they do not have any conflict of interest.

## AUTHOR CONTRIBUTION

**Narjes Aghajani:** Conceptualization (equal); Supervision (equal); Writing – original draft (equal); Writing – review & editing (equal). **Ashraf Gohari Ardabili:** Supervision (equal); Validation (equal). **Fahime Ahmadi:** Data curation (equal); Formal analysis (equal).

## ETHICAL APPROVAL

This study does not involve any human or animal testing.

## INFORMED CONSENT

Written informed consent was obtained from all study participants.
